# Burnout among psychotherapists: a cross-cultural value survey among 12 European countries during the coronavirus disease pandemic

**DOI:** 10.1038/s41598-022-17669-z

**Published:** 2022-08-08

**Authors:** Angelika Van Hoy, Marcin Rzeszutek, Małgorzata Pięta, Jose M. Mestre, Álvaro Rodríguez-Mora, Nick Midgley, Joanna Omylinska-Thurston, Anna Dopierala, Fredrik Falkenström, Jennie Ferlin, Vera Gergov, Milica Lazić, Randi Ulberg, Jan Ivar Røssberg, Camellia Hancheva, Stanislava Stoyanova, Stefanie J. Schmidt, Ioana Podina, Nuno Ferreira, Antonios Kagialis, Henriette Löffler-Stastka, Ewa Gruszczyńska

**Affiliations:** 1 Faculty of Psychology, University of Warsaw, Stawki 5/7, 00-183, Warsaw, Poland; 2 University Institute of Sustainability and Social Development (INDESS), Jerez de la Frontera, Universidad de Cádiz, Department of Psychology, Puerto Real (Cádiz), Spain; 3 Child Attachment and Psychological Therapies Research Unit (ChAPTRe), Anna Freud Centre for Children and Families, 4-8 Rodney Street, London, N1 9JH UK; 4 School of Health and Society, University of Salford, Frederick Road Campus, Broad Street, Salford, M6 6PU UK; 5 School of Psychology, University of Lincoln, Brayford Pool, Lincoln, LN6 7TS UK; 6 Department of Psychology, Linnaeus University, SE-351 95 Växjö, Sweden; 7 Linköping University, 581 83 Linköping, Sweden; 8 Department of Psychology and Logopedics, Faculty of Medicine, University of Helsinki, P.O. Box 63, 00014 Helsinki, Finland; 9 Department of Psychology, Faculty of Philosophy, University of Novi Sad, 2 Dr Zorana Đinđića, 21101 Novi Sad, Serbia; 10 Insitute of Clinical Medicine, University of Oslo, Blindern, P.O Box 1171, 0315 Oslo, Norway; 11 Department of Psychology, Head of Center for Psychological Counselling and Research, Sofia University “St. Kliment Ohridski, 15 Tsar Osvoboditel Blvd., 1504 Sofia, Bulgaria; 12 Department of Psychology, Faculty of Philosophy, South-West University “Neofit Rilski”, 66 Ivan Mihaylov Street, 2700 Blagoevgrad, Bulgaria; 13 Department of Clinical Child and Adolescent Psychology, Institute of Psychology, University of Bern, Fabrikstrasse 8, 3012 Bern, Switzerland; 14 Faculty of Psychology and Educational Sciences, University of Bucarest, 90 Panduri Street, sector 5, 050663 Bucharest, Romania; 15 Department of Social Sciences, School of Humanities and Social Sciences, University of Nicosia, 46 Makedonitissas Avenue, 2417 Nicosia, Cyprus; 16 Department of Psychiatry, School of Medicine, University of Crete, 715 00 Heraklion, Greece; 17 Department of Psychoanalysis and Psychotherapy, Medical University Vienna, Währinger Gürtel 18-20, 1090 Vienna, Austria; 18 Faculty of Psychology, SWPS University of Social Sciences and Humanities, Chodakowska 19/31, 03-815 Warsaw, Poland

**Keywords:** Psychology, Human behaviour, Quality of life

## Abstract

The aim of this study was to examine cross-cultural differences, as operationalized by Schwartz's refined theory of basic values, in burnout levels among psychotherapists from 12 European countries during the coronavirus disease (COVID-19) pandemic. We focused on the multilevel approach to investigate if individual- and country-aggregated level values could explain differences in burnout intensity after controlling for sociodemographic, work-related characteristics and COVID-19-related distress among participants. 2915 psychotherapists from 12 countries (Austria, Bulgaria, Cyprus, Finland, Great Britain, Serbia, Spain, Norway, Poland, Romania, Sweden, and Switzerland) participated in this study. The participants completed the Maslach Burnout Inventory-Human Service Survey, the revised version of the Portrait Values Questionnaire, and a survey questionnaire on sociodemographic, work-related factors and the COVID-19 related distress. In general, the lowest mean level of burnout was noted for Romania, whereas the highest mean burnout intensity was reported for Cyprus. Multilevel analysis revealed that burnout at the individual level was negatively related to self-transcendence and openness-to-change but positively related to self-enhancement and conservation values. However, no significant effects on any values were observed at the country level. Male sex, younger age, being single, and reporting higher COVID-19-related distress were significant burnout correlates. Burnout among psychotherapists may be a transcultural phenomenon, where individual differences among psychotherapists are likely to be more important than differences between the countries of their practice. This finding enriches the discussion on training in psychotherapy in an international context and draws attention to the neglected issue of mental health among psychotherapists in the context of their professional functioning.

## Introduction

Since Freud's^1^ early observation of the *danger of analysis for the analyst*, subsequent empirical studies have shown that psychotherapists may be vulnerable to burnout (see reviews and metanalyses^2,3^. Although this highly emotionally taxing helping profession should be a textbook example of a job with a high risk of burnout^4–7^, studies on burnout among psychotherapists are much less prevalent than those on burnout in other similar health professions such as physicians or nurses (see reviews and meta-analyses^8–12^). Thus, the issue of burnout in this occupation was and is still largely understudied in the fields of clinical psychology and psychotherapy, which are traditionally focused on the clients of psychotherapy rather than on psychotherapists^13,14^. However, several authors have observed that burned-out psychotherapists not only lose their ability to maintain their therapeutic relationship with clients and manage the whole therapeutic process^15–18^ but also experience a substantial decline in their well-being, accompanied by various somatic and psychological complaints^19–21^. Until now, the most commonly studied burnout risk factors among psychotherapists were either work-related (e.g., caseload and years of experience) or sociodemographic (sex and age)^3^. Much less attention was paid to the interpersonal and intrapersonal characteristics of therapists^22^. In addition, several studies found vast discrepancies in burnout prevalence among psychotherapists from various countries, ranging from 6%–54%^3^. To date, however, the cultural context has only been examined from the perspective of client outcomes, not how it potentially relates to a psychotherapist's functioning and well-being^23^. In our study, we followed the basic cultural values in the refined Schwartz value theory^24,25^ to assess burnout differences among psychotherapists from 12 European countries during the coronavirus disease (COVID-19) pandemic. To combine both individual and cultural perspectives, we employed the multilevel approach, which allowed us to evaluate burnout at different levels of this hierarchy simultaneously^26,27^. This approach may provide new insight into the fundamental question of whether burnout is a multidimensional phenomenon or unitary, single-factor syndrome consisting of interrelated symptoms^27^. In our study, we wanted to verify whether, among psychotherapists from these 12 countries, differences in professional burnout were associated with individual and country-aggregated Schwartz’s values, after controlling for sociodemographic and work-related characteristics as well as the COVID-19 distress.

According to Schwartz^28^, values are “desirable trans-situational goals, varying in importance, that serve as guiding principles in the life of a person or other social entity.” The recently refined theory of basic values^24,25^ highlights 19 basic values, which can be grouped into four higher-order values (self-transcendence, self-enhancement, openness to change, and conservation; see Measures section). These values were recognized in all major cultures^29^ and are associated distinctively with human attitudes, behaviors, and demographic variables. The main important assumption in this theory relates to a circular motivational continuum of values, which shows motivational conflict or compatibility across distinct values^30,31^. In other words, values can be compatible if decisions and behaviors that express the goals of one value also correspond to the goals of the other value. By contrast, values conflict if decisions or behaviors that express the goals of some values do so at the cost of other values. However, one of the still unresolved research questions is to what extent one may observe high within-country similarity and significant between-country variability in the culture as a shared meaning system^32–34^. This problem becomes even more interesting if there is a mismatch between individual values declared by a citizen of a particular country and a country-aggregated level of these values^32^. For example, Stephens et al.^35^ observed that a culturally mismatched environment can be associated with significant psychological distress, which can even impact the biological functioning of the person experiencing such mismatch. In the issue of psychotherapists, existing reviews and meta-analyses have revealed that the link between burnout and work-related factors may be modified by cultural differences, which shape not only the organizational characteristics of this profession but even the types of therapeutic relationships formed with clients^2,3,9^. Nevertheless, those cultural factors have never been explicitly measured in previous studies. Therefore our study is the first to apply a well-established theoretical model to interpersonal and cross-cultural comparisons of values and their potential impact on burnout among psychotherapists. Finally, we also took into account the most recent and thus, much understudied potential burnout risk factor among psychotherapists, which is the psychological distress during the COVID-19 pandemic^36,37^. In light of the COVID-19 pandemic, psychotherapists were faced with many new challenges and obstacles regarding their therapeutic practice. Many psychotherapists either stopped working altogether or changed their practices in some form. One of the main challenges encompassed switching entirely or partly to providing psychotherapy in an online format. The above-mentioned factors were responsible for elevated levels of depression, anxiety and loneliness in this particular sample^36^. However, till now no studies have been conducted on how the COVID-19 pandemic could be related to burnout among psychotherapists employing cross-cultural comparisons.

## Present Study

The main aim of this study was to examine the cross-cultural differences in burnout intensity among psychotherapists from 12 countries during the COVID-19 pandemic. We focused on the multilevel approach to investigate if individual- and country-aggregated level values, as operationalized by Schwartz's refined theory of basic values^24,25^, could explain differences in burnout after controlling for sociodemographic and work-related characteristics and COVID-19-related distress. We formulated the following hypotheses at the individual and country-aggregated levels to determine whether burnout in that specific occupation is more an individual syndrome or mostly shaped by the between-country differences in values declared by psychotherapists. To the best of our knowledge, no studies have been conducted on burnout in this group of participants using such a concrete model of culture and methodological design. Thus, our study is mainly explorative.

### Hypothesis 1

Burnout among psychotherapists is significantly related to individual-level values (self-transcendence, self-enhancement, openness to change, and conservation) after controlling for sociodemographic and work-related characteristics and COVID-19-related distress.

### Hypothesis 2

Burnout among psychotherapists is significantly related to country-aggregated values (self-transcendence, self-enhancement, openness to change, and conservation) after including all the variables from Hypothesis 1.

### Hypothesis 3

Burnout among psychotherapists is related to cross-level interactions in a way that a higher level of burnout is associated with a higher mismatch between the values declared at the individual- and country-aggregated levels.

## Methods

### Participants

We conducted a cross-cultural survey using standardized questionnaires in online format (see below) via the specialized survey platform among psychotherapists from 12 European countries: Austria, Bulgaria, Cyprus, Finland, Great Britain, Serbia, Spain, Norway, Poland, Romania, Sweden, and Switzerland. The data collection in all the countries was parallelly conducted between June 2020 and June 2021, during the second and third waves of the COVID-19 pandemic. The online set of the study questionnaires was sent in each country to the professional psychotherapeutic associations of various therapeutic modalities, which have distributed it among their members.

Finally, 2915 psychotherapists from the 12 countries representing various psychotherapeutic modalities participated in this study. The eligibility criteria encompassed certification (or being in the process of certification) in a particular psychotherapeutic modality and psychotherapeutic practice for at least 1 year. The participants completed the online versions of the questionnaires, which were preceded by detailed sociodemographic and work-related questions, including items on how the COVID-19 pandemic impacted their practice and on potential psychological distress associated with the pandemic. In each country, participation was anonymous and voluntary, and the participants received no remuneration for participating in the survey. Informed consent was obtained from all participants of this study. The study protocol was accepted by the ethics committee of the Faculty of Psychology at the University of Warsaw in Poland. The sociodemographic and work-related variables and COVID-19-related distress among the psychotherapists from each country are presented in the Supplementary Tables. Finally, it is important to underline that this manuscript contains unique data, which has not been published in any other journal.

As can be seen in all the tables, age distributions were generally similar among all countries (M = 45.5 years, min. 21 years—max. 82 years). Regarding the participants' sexes, female psychotherapists were overrepresented (83%) in all of the countries. A significant number of participants were also in some form of stable relationships (75%). In terms of education, most participants held psychology degrees. However, Finnish and Swedish participants were almost evenly divided between having a psychology degree or a different degree such as social work, counseling, or nursing. In all 12 countries, most psychotherapists worked with adult clients. Nonetheless, a significant number of Polish and Bulgarian psychotherapists also worked with children. Having a private workplace was almost universal for therapists in all countries. Most psychotherapists in all the countries had already undergone their own psychotherapy. Supervision was provided once a month to the participants in most of the countries. However, Austrian psychotherapists used supervision quarterly, and most Spanish therapists did not use it at all. The results regarding therapeutic modalities varied across countries. Cognitive-behavioral therapy seemed to be the more common therapeutic approach in Cyprus, Spain, Poland, and Romania. Next, psychodynamic therapy was the dominant modality in Bulgaria, Norway, and Sweden. Austria and Switzerland seemed to favor Gestalt therapy. Finally, integrative psychotherapy was the most common approach in the United Kingdom. On average, psychotherapists in Bulgaria, Cyprus, Finland, Poland, Romania, and Serbia had between 6 and 11 years of experience in the profession. On the other hand, psychotherapists who were working in Austria, Spain, Norway, Switzerland, Sweden, and the United Kingdom had between 12 and 18 years of experience. In eight of the included countries (Austria, Cyprus, Finland, Spain, Norway, Romania, Switzerland, and the United Kingdom), most psychotherapists reported having a psychology certification (80% or more). The numbers appeared lower in Bulgaria, Poland, Serbia, and Sweden, with only approximately 35–65% of psychotherapists obtaining a certificate. Psychotherapists worked anywhere between a couple of hours a week and more than 20 h a week. More specifically, the average weekly workload in Bulgaria, Cyprus, Romania, and Serbia was between 1 and 10 h. In Sweden and the United Kingdom, the average was between 10 and 20 h a week. Psychotherapists who worked for more than 20 h a week were from Finland, Norway, and Poland. Austrian, Spanish, and Swiss psychotherapists were evenly divided between the last two workload categories. Finally, a general trend in working partially online during the COVID-19 pandemic was observed, with this being the case for psychotherapists in 11 countries (Austria, Bulgaria, Cyprus, Finland, Spain, Norway, Poland, Romania, Serbia, Switzerland, and Sweden). At the time of data collection, UK therapists were still mostly providing their services online only.

### Measures

To assess burnout, we used the Maslach Burnout Inventory-Human Service Survey (MBI-HS)^6^. All 12 language adaptations of the MBI-HS were bought from Mind Garden, the official distributor of the MBI-HS. The MBI-HS consists of 22 items and evaluates burnout and its three components: (1) Emotional Exhaustion (EE), nine items; (2) Personal Accomplishment (PA), eight items; and (3) Depersonalization (DP), five items. For each item, the respondent indicated the frequency of symptoms on a Likert-type scale from 0 (never) to 6 (every day). ). All the summed responses form an overall index, higher values of which indicate higher burnout. We decided to use the MBI-HS in our study for two reasons: First, it is the most popular and widely used burnout inventory focused especially on helping professions, which was the case in our research^7,38^. Second, the MBI-HS is the only tool available for the assessment of burnout with a wide spectrum of different language adaptations; as such, it is valuable in cross-cultural studies^38^.

To measure cultural values, the participants completed a revised version of the Portrait Values Questionnaire (PVQ-R) developed by Schwartz et al.^24^. The PVQ-R consists of 57 short, sex-matched, verbal portraits of different people, each depicting a goal that is important to some person. For each portrait, respondents highlight how similar the person is to themselves on a 6-point Likert-type scale defined as follows: 1—not like me at all, 2—not like me, 3—a little like me, 4—moderately like me, 5—like me, and 6—very much like me. The participants' values are inferred from the values of the other people they described as similar to themselves. For example, a respondent who underlines that a person described as "Enjoying life's pleasures is important to her" is similar to herself, and probably attributes importance to hedonistic values. The PVQ-R assesses 19 values that can be combined into higher-order values, which was the case in our study: self-transcendence (universalism-nature, universalism-concern, universalism-tolerance, benevolence-care, and benevolence-dependability), self-enhancement (achievement, power dominance, and power resources), openness to change (self-direction thought, self-direction action, stimulation, and hedonism), conservation (security-personal, security-societal, tradition, conformity-rules, and conformity-interpersonal). All the language versions of the PVQ-R were provided by the author of this tool, S. Schwartz.

COVID-19 related distress was assessed via short, but reliable operationalization of this variable based on some other studies published at the time, when we started our research^39,40^. Namely, we asked participants on a Likert 1–5 point scale how stressful they found the situation in their role as psychotherapists caused by the COVID-19 pandemic. The answers varied between 1 (“not at all)” to 5 (“very much”). We also examined the issue of changes in psychotherapy settings (i.e. online setting) imposed by the pandemic situation.

### Data analysis

The data obtained had a two-level structure with persons (2915 units) nested within countries (12 units); thus, a cross-sectional multilevel model was adopted^41^. The explained variable was the burnout level among the psychotherapists, which was operationalized as the global burnout indicator. The explaining variables at Level 1 were the four higher-order values assessed by each person (see Measures section), centered on their means (centering on the group mean). The Level 2 variables were aggregates of the individual person's scores on four higher-order values to form a country mean of each value, which was then centered on the mean for all countries at a given value (see, centering on the grand mean). The maximum likelihood (ML) estimation method was used. For random effects (the random intercept model), the covariance structure of the variance components (VC) was assumed.

Unconditional (i.e., intercept only) modeling was the first step of the analysis. It was also used to obtain the interclass correlation coefficient (ICC)^42^, which informs about the proportion of variance in the burnout level explained by a grouping variable, that is, a country in which a participant is a psychotherapist. ICC values as low as 0.01 were treated as non-trivial^43^. Next, sociodemographic and work-related characteristics and COVID-19-related distress were added to the model. Continuous variables were centered on the group mean (e.g., age, work experience, and pandemic-related stress), whereas categorical variables were transformed into two dummy-coded categories (sex: female = 0, male = 1; relationship status: single = 0, in a stable relationship = 1; weekly workload: 0 = less than 20 h, 1 = 20 h and more; supervision: 0 = quarterly or less, 1 = once a month or more). In subsequent steps, only the variables found to be significantly related to the explained variable were taken into account^44^. In the third step, the Level 1 personal values were added, followed by the introduction of the Level 2 aggregates of these values for each country in the fourth step. Finally, the cross-level interactions of all values were tested^45,46^. For significant cross-level interactions, simple slopes, regions of significance, and confidence bands were established using the computational tools developed by Preacher et al.^47^. Statistical analysis was performed using IBM SPSS Statistics version 27^48^. Only the final hypothesis-testing models are presented in the article.

For model comparison, deviance statistics, based on *χ*^2^ distribution with the degrees of freedom equal to the difference in the number of parameters estimated in nested models, and the Akaike Information Criterion were used^41^.

### Ethical approval

All procedures performed in studies involving human participants were in accordance with the ethical standards of the institutional and/or national research committee and with the 1964 Helsinki Declaration and its later amendments or comparable ethical standards.

## Results

### Descriptive statistics

Table 1 presents descriptive statistics on burnout levels and the four higher-order values for each national sample of therapists.

**Table 1:** Descriptive statistics for overall burnout level and personal values in the study sample of psychotherapists (N = 2915) according to country of origin.

Country	n	Mean	SD	Range	Kurtosis	Skewness
**Burnout**						
Austria	151	32.21	14.26	9–76	0.89	1.05
Bulgaria	217	38.36	15.26	8–105	0.97	0.79
Cyprus	202	42.50	17.74	10–85	− 0.72	0.47
Finland	254	31.32	12.52	8–82	0.84	0.89
Norway	225	42.12	16.27	11–89	0.13	0.60
Poland	340	37.38	14.28	10–122	4.19	1.22
Romania	202	26.50	13.51	8–74	0.73	1.06
Serbia	237	31.39	14.28	8–79	0.68	0.89
Spain	320	35.61	16.03	8–90	0.41	0.85
Sweden	275	40.53	16.00	9–106	0.61	0.74
Switzerland	205	33.90	12.95	9–74	0.44	0.77
United Kingdom	287	42.14	17.19	10–103	0.46	0.76
**Self-transcendence**						
Austria	150	4.83	0.77	1–6	1.49	− 1.17
Bulgaria	217	4.48	0.79	1–6	− 0.80	1.19
Cyprus	202	4.91	0.65	2–6	1.32	− 0.93
Finland	254	4.86	0.55	3–6	− 0.66	0.05
Norway	225	4.82	0.63	1–6	3.23	− 1.20
Poland	340	5.01	0.50	3–6	1.03	− 0.64
Romania	202	4.82	0.68	1–6	5.43	− 1.63
Serbia	237	4.38	0.64	2–6	1.91	− 1.13
Spain	320	5.09	0.55	3–6	0.88	− 0.88
Sweden	275	4.83	0.57	3–6	− 0.52	− 0.40
Switzerland	205	5.10	0.54	3–6	0.00	− 0.63
United Kingdom	287	4.90	0.58	2–6	0.67	− 0.73
**Self-enhancement**						
Austria	151	3.02	0.75	1–6	− 0.47	0.25
Bulgaria	217	3.14	0.81	1–6	− 0.46	0.22
Cyprus	202	3.64	0.85	1–6	− 0.14	− 0.27
Finland	254	2.67	0.89	1–6	0.46	0.84
Norway	225	3.29	0.93	1–6	− 0.44	0.44
Poland	340	2.88	0.83	1–6	0.50	0.62
Romania	202	3.37	0.81	1–6	0.70	0.11
Serbia	237	3.71	0.69	2–6	0.13	0.22
Spain	320	2.88	0.80	1–6	0.42	− 0.22
Sweden	275	2.88	0.85	1–6	0.16	0.78
Switzerland	205	2.94	0.76	1–5	− 0.31	0.39
United Kingdom	287	3.24	0.74	1–6	− 0.30	0.28
**Openness to change**						
Austria	151	4.53	0.73	2–6	0.39	− 0.58
Bulgaria	217	4.31	0.79	1–6	0.64	− 0.47
Cyprus	202	3.64	0.66	2–6	0.74	− 0.54
Finland	254	3.98	0.60	2–6	− 2.94	− 0.22
Norway	225	4.25	0.62	1–6	− 0.82	2.56
Poland	340	4.56	0.57	2–6	0.28	− 0.37
Romania	202	4.63	0.69	1–6	3.43	− 0.99
Serbia	237	4.42	0.70	2–6	0.96	− 0.92
Spain	320	4.76	0.56	2–6	0.07	− 0.35
Sweden	275	4.26	0.59	2–6	− 0.31	0.11
Switzerland	205	4.74	0.58	2–6	0.12	− 0.50
United Kingdom	287	4.41	0.63	2–6	− 0.19	− 0.23
**Conservation**						
Austria	151	3.66	0.65	2–6	− 0.07	− 0.04
Bulgaria	217	3.79	0.75	1–6	0.78	− 0.21
Cyprus	202	4.31	0.70	1–6	1.10	− 0.61
Finland	254	3.77	0.72	2–6	− 0.57	0.00
Norway	225	3.89	0.72	1–6	0.35	− 0.42
Poland	340	3.69	0.70	1–6	− 0.50	0.31
Romania	202	3.91	0.66	1–6	1.81	− 0.84
Serbia	237	4.28	0.66	2–6	0.55	− 0.60
Spain	320	3.98	0.80	1–6	− 0.13	− 0.20
Sweden	275	3.57	0.71	1–6	− 0.21	0.21
Switzerland	205	3.69	0.67	1–6	− 0.39	− 0.02
United Kingdom	287	3.70	0.73	1–6	− 0.51	0.05

**Figure 1:**
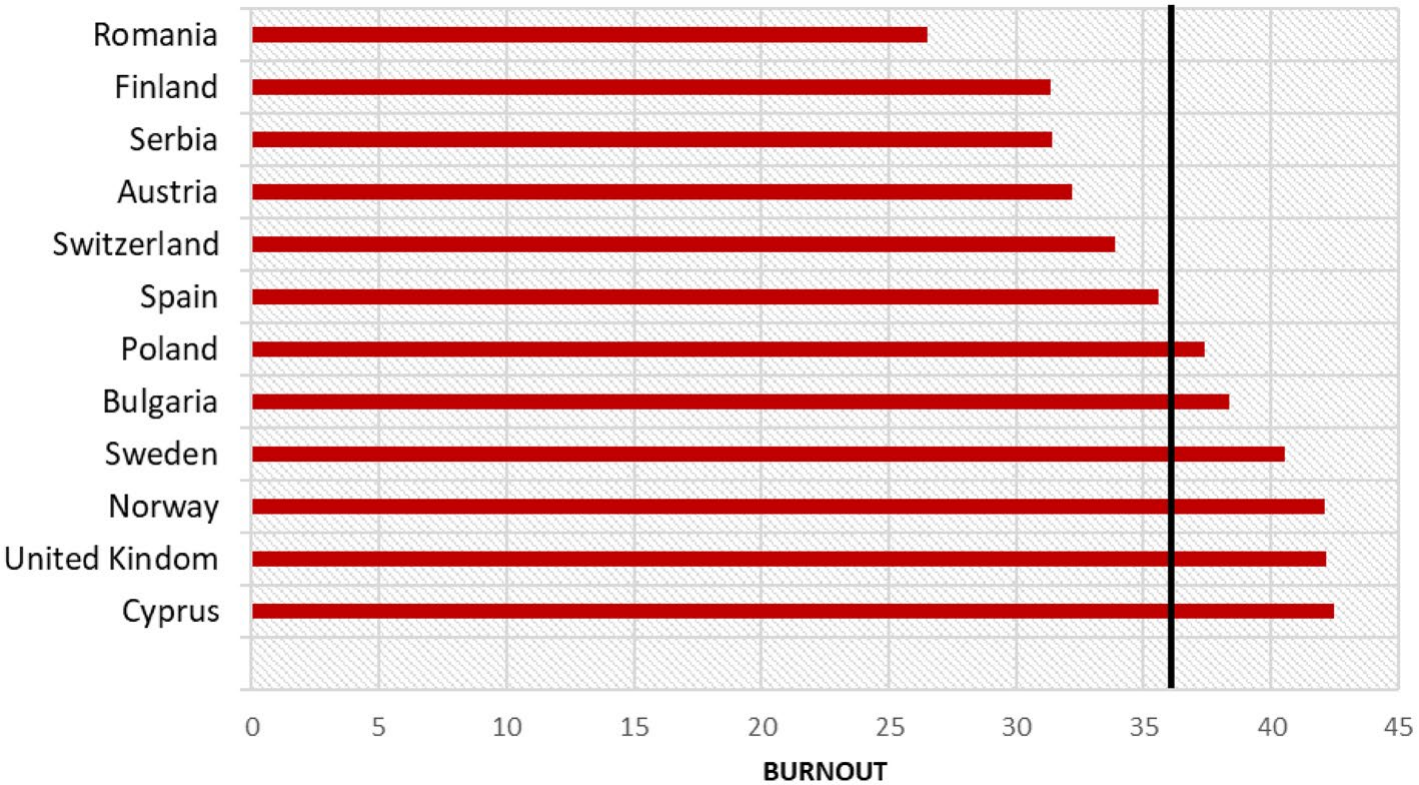
Burnout mean levels values per country. A black line indicates a grand mean.

Figure 1 illustrates the mean burnout levels at the country level. The lowest mean was noted for Romania, whereas the highest mean was reported for Cyprus. However, the ICC equals 0.09; thus, only 9% of the variance of burnout level in the study sample of psychotherapists was related to the country level.

### Hypothesis testing

The results of the hypothesis testing are presented in Table 2.

**Table 2:** Results for the hypothesis testing for the overall burnout indicator in the study sample of psychotherapists (N = 2915).

	Hypothesis 1 model		Hypothesis 2 model		Hypothesis 3 model	
	Estimate (SE)		Estimate (SE)		Estimate (SE)	
**Fixed effects**						
Intercept	37.11	(1.50)***	37.10	(1.31) ***	37.14	(1.35) ***
Sex	1.70	(0.69)**	1.68	(0.69) **	1.68	(0.69) ***
Relationship status	− 1.54	(0.61)**	− 1.54	(0.61) **	− 1.58	(0.61) **
Age	− 0.33	(0.03)***	− 0.33	(0.03) ***	− 0.33	(0.03) ***
COVID-19-related stress	4.33	(0.26)***	4.33	(0.26) ***	4.33	(0.26) ***
Level 1 values						
Self-transcendence_w	-4.01	(0.56)***	− 4.01	(0.56)***	− 4.10	(0.56)***
Self-enhancement_w	1.65	(0.39)***	1.65	(0.39)***	1.61	(0.39)***
Openness to change_w	− 2.52	(0.52)***	− 2.52	(0.52)***	− 2.57	(0.52)***
Conservation_w	0.94	(0.45)**	0.94	(0.45)**	0.92	(0.45)**
Level 2 values						
Self-transcendence_b			15.88	(9.71)	15.89	(9.71)
Self-enhancement_b			12.84	(7.82)	12.84	(7.82)
Openness to change_b			− 10.66	(7.81)	− 10.66	(7.81)
Conservation_b			− 6.42	(8.69)	− 6.43	(8.69)
Cross-level interactions						
Self-transcendence_w*b					− 0.97	(2.08)
Self-enhancement_w*b					1.80	(1.14)
Openness to change_w*b					− 3.81	(1.92)**
Conservation_w*b					− 0.06	(1.82)
**Random effects**						
Residual variance	185.20	(4.95)***	185.21	(4.94)***	184.71	(4.93)***
Between-country variance (intercept)	22.32	(9.49)**	17.04	(7.35)**	17.04	(7.35)**
**Model parameters**						
Akaike Information Criterion	22,765.39		22,770.33		22,770.85	
− 2LL	22,743.39		22,740.33		22,732.85	
− 2 LL Δ (df)			3.06 (4)		7.48 (4)	

***p < 0.001, **p < 0.05.

For Hypothesis 1, the test revealed that the psychotherapist-reported self-transcendence and openness-to-change values that were higher than the typical values for a national sample were related to the lower overall burnout levels of the psychotherapists. On the other hand, the higher-than-typical self-enhancement and conservation values were related to higher overall burnout levels. Moreover, we observed significant associations of burnout with some of the sociodemographic and work-related characteristics as well as COVID-19-related distress. The burnout correlates were male sex, being single, younger age, and reporting more intense pandemic-related stress than typical for the national sample.

For Hypothesis 2, after controlling for all the variables mentioned in Hypothesis 1, the differences in values at the country-aggregated level were not significant for burnout.

**Figure 2:**
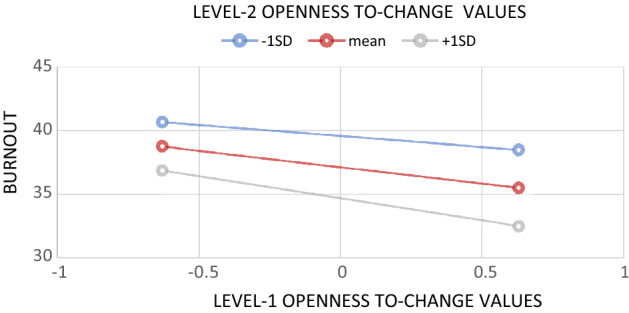
Simple slopes for cross-level interaction for openness-to-change values on burnout. For Level-2 openness-to-change values slopes are probed at a mean and one standard deviation above and below a mean. Level-1 openness-to-change values were centered around within-country means.

Finally, with regard to Hypothesis 3, we observed significant cross-level interaction between openness-to-change values reported at individual- and aggregated country-level (*B* =  − 3.81, SE = 1.92, t = 1.98, *p* < 0.05). The analysis of simple slopes is presented in Fig. 2. As can be observed, the openness-to-change values were more negatively related to burnout among psychotherapists in the countries with aggregated openness-to-change values higher than the cross-country average (*Β* =  − 3.47, SE = 0.70, z =  − 4.98, *p* < 0.001) in comparison to the countries for which these aggregated values were lower (*Β* =  − 1.72, SE = 0.68, z =  − 2.55, *p* < 0.05). Thus, the protective effect of being individually highly localized on openness-to-change values in the national sample was further amplified by originating from a country with aggregated openness-to-change values higher than the average for all 12 studied countries. Referring these results to the confidence bands of the aggregated values (− 61.83, − 0.29), inside which the simple slopes were equal to zero, we conclude that there was no relationship between personal openness-to-change values and burnout only for psychotherapists from Finland (*Β* =  − 0.75, SE = 1.05, z =  − 0.73, *ns*), which at the country level has the lowest openness to change among the studied countries. However, this result should be interpreted with caution as a model including interaction is not significantly better fitted to the data than a model including only main effects.

## Discussion

The results of our study were in accordance with our hypotheses at the individual level rather than at the country-aggregated level of analysis. At the individual level, burnout was negatively related to the self-transcendence and openness-to-change values but positively related to the self-enhancement and conservation values. Although Schwartz's^30^ theory of basic human values has been used in hundreds of studies and various theoretical contexts^24,25,49^, it has not been applied to the issue of psychological disorders. Owing to the fact that this is the first study to link cross-cultural values to burnout syndrome among psychotherapists, this result is difficult to discuss other than exploratorily. Nevertheless, it is intriguing that motivational goals expressed in higher-order values of self-enhancement (e.g. power-dominance) and conservation (tradition, conformity-rules) were found to be burnout predictors, while motivational goals of self-transcendence (e.g. universalism-tolerance) and openness-to-change (e.g. self-direction thought) acted as buffers against burnout in this particular sample. Thus, our study may be an interesting adjunct to the literature on the psychological functioning of psychotherapists, including the neglected cross-cultural context^2,3^. As part of the psychotherapy training process, it is also important to consider this latter context.

However, the most intriguing finding was somehow a null result at the country-aggregated level. At this level, differences in values were irrelevant to the burnout levels of the participants. This was also confirmed by the comparisons of the effects at the individual and country levels. For example, we observed differences in burnout among the 12 countries, with the highest levels in Cyprus, Sweden, Norway, and the United Kingdom and the lowest levels in Romania, Serbia, and Finland. Nevertheless, these differences were explained almost entirely by interpersonal differences, as only 9% of the burnout variance was related to the country level. From a different perspective, burnout among psychotherapists tends to be a *transcultural phenomenon* rather than a country-specific problem. Although values do matter, their idiosyncratic aspect is more important for burnout than the collectivist aspect, i.e. shared by a group of representatives of this profession in a given country. This may be an important conclusion for reflection on the organizational structure and training in psychotherapy in Europe^13,50^.

The country-level aggregated values were found to be significant in the only observed cross-level interaction concerning openness to change. This supports the hypothesis on the role of fit between individual and collective values^24,25^. Namely, the protective effect of individual values was enhanced when being a psychotherapist in a country where other psychotherapists also declared high openness-to-change values. However, this result requires further research. Observing it only for this category of values may in fact be due to the specific circumstances of the study. The COVID-19 pandemic universally enforced adaptation to the "new normal". Burnout may therefore actually affect to a lesser extent those who consider openness to change as an important value in their lives since they have an intrinsic motivation for novelty and mastery, but this adaptation may also be facilitated or hindered by what happens in the social environment of such a person. The attitudes represented by one's occupational group, especially when the external demands include major changes in the conditions of work, are likely to become an influential reference point to modify an individual's appraisals and behaviors.

We found that higher burnout levels among psychotherapists were associated with sociodemographic data (younger age, being single, and male sex) and higher levels of COVID-19-related distress. Previous studies on burnout among psychotherapists have shown that younger psychotherapists are at greater risk of burnout than older psychotherapists and usually more experienced colleagues^15,51–53^. This finding is often explained by the fact that young psychotherapists may have high and unrealistic expectations about their roles in this occupation, and a subsequent *reality crash* may be a burnout catalyst^52^. Our study also showed that male psychotherapists can be at a higher risk of burnout than female psychotherapists, but the results reported in the literature on this topic are discrepant^54,55^. Our meta-analysis revealed that men and women may experience burnout in different ways; for example, women score higher on emotional exhaustion, whereas men score higher on depersonalization^56^. Consistent with our findings, the psychotherapy profession may also be associated with burnout among men due to sex-related differences in self-efficacy, which is usually higher among females in helping professions^57^. As expected, COVID-19-related distress was a significant burnout correlate in all the countries included in the study, which is consistent with the most recent research^36,37^. However, this subject is still understudied in general, particularly in this sample. In light of the COVID-19 pandemic, psychotherapists were faced with many new challenges and obstacles regarding their practices, clients, and their well-being.

In a more general discussion, our findings contradict one of the main assumptions at the root of cross-cultural psychology, which is high within-country similarity and significant between-country variability in shared cultural meaning systems^33,34,58^. This notion suggests conducting cross-country comparisons by “unpacking” cultural differences within the studied psychological constructs and discussing them in light of a culture-comparative perspective^33^. Nevertheless, for at least two decades, attempts have been made to calculate the effect sizes of the aforementioned within-culture consensus and cross-cultural variability in several theoretical constructs^59,60^. Fisher and Schwartz^32^ examined values in 67 countries and observed negligible variances in value ratings that may be associated with country differences. Specifically, they found that the cross-country differences and within-country consensus in values were very low in all examined countries. Thus, we can infer that this is not because values are part of some shared meaning system defined as culture but because people, in general, differ in values regardless of where they come from. We obtained a similar pattern of results in this study. However, the aforementioned problem needs further examination, as we did not observe a consistent pattern of the effects of a mismatch between individual and country-aggregate values on burnout outcomes at the cross-level interactions (Fig. 2). The clinical context in cross-cultural psychology, that is, the role of values in psychological disorders, is, therefore, an important research gap to address in the future.

### Strengths and Limitations

This study has several strengths, including its large sample of psychotherapists from 12 different countries observed during the critical period of the COVID-19 pandemic and the use of a theoretical model for cross-cultural comparisons and a multilevel design, which make it a pioneer study in the relevant literature. However, several limitations should be mentioned. First, for organizational reasons, our samples of psychotherapists were heterogeneous concerning psychotherapeutic modalities and other work-related characteristics. In addition, they cannot be considered representative of the countries in which they were sampled. This represents a common shortcoming in the literature on the psychological functioning of this professional group^3^ but is hard to avoid, particularly in international comparisons and associated differences in regulations for this job between countries. Second, our research shares other common limitations in burnout studies among psychotherapists, including its cross-sectional design and precluding causal inferences^2^. The role of values in the prospective study design would be interesting to investigate to determine the stability of its effect at the individual and country levels. Two typical shortcomings must be borne in mind in cross-cultural research, namely the reference group effect^61^ and the response style effect^62^. The former deals with the problem of using a self-report measure to assess cross-cultural differences when participants compare themselves to familiar others (e.g., Poles compared themselves to other known Poles). The latter illustrates culture-related differences in response styles. These effects may also be the reason for the small effects of the country-aggregated level of analysis. However, another reason may be that there are not enough units of analysis at this level. A general rule of thumb is to have as many units at a higher level as possible^63^. In cross-cultural research, because of practical considerations, this rule is rarely fulfilled^64^. Finally, our study was not limited in terms of the number of countries, but also in terms of their location. The participants represented only European countries, which was due both to organizational issues, including the course of the pandemic, but also to the sharing of the basic foundations of psychotherapy as a profession. Future research should therefore focus on comparisons that cover a broader spectrum of countries.

## Conclusions

In light of the recent inclusion of burnout in the 11th Revision of the International Classification of Diseases^65^, one should bear in mind that burnout is a global occupational phenomenon that can be observed in any profession^10^, including psychotherapists^3^. Our data suggest that burnout among psychotherapists may be, in some sense, a transcultural phenomenon, in which there is room for interplay between what is individual and what is shared with one's occupational group. However, the most important factors are the individual differences between psychotherapists, regardless of their cultures, at least across the studied European countries. Although this finding should be treated with caution because of the explorative characteristics and limitations of our study, it may be an enriching adjunct to the discussion on preventing psychotherapists’ burnout. Specifically, the results of our study call for the need to place more focus on psychotherapists’ personal values regarding their professional and private lives, especially during the psychotherapy training process. It has been found that these are crucial factors that promote the personal and professional quality of life in this profession^66^.

## Supplementary Information

Supplementary Information 1.

Supplementary Information 2.

Supplementary Information 3.

## Data Availability

All data generated or analysed during this study are included in this published article and its supplementary information files.
