# Supplementary material for: Burnout among psychotherapists: a cross-cultural value survey among 12 European countries during the coronavirus disease pandemic
Source: Sci Rep. 2022 Aug 8;12:13527. doi: 10.1038/s41598-022-17669-z (PMC9358385; doi:10.1038/s41598-022-17669-z)
Supplement: Supplementary file 2 — Supplementary Information 2. [file 41598_2022_17669_MOESM2_ESM.docx]

Table 1. *Sociodemographic, work-related, and COVID-19-related stress data of the psychotherapists from Austria (N = 151).*

|  | n | % |  |  | n | % |
| --- | --- | --- | --- | --- | --- | --- |
| Sex |  |  |  | Clients |  |  |
| Male | 40 | 26.5 |  | Children/youth | 60 | 39.7 |
| Female | 111 | 73.5 |  | Adults | 145 | 96.0 |
| Age in years (mean ± SD) | 51.28 | 10.24 |  | Couples | 39 | 25.8 |
| Marital status |  |  |  | Families | 59 | 39.1 |
| Stable relationship | 114 | 75.5 |  | Workplace |  |  |
| Divorced/separated | 14 | 9.3 |  | Public | 14 | 9.3 |
| Widow/widower | 1 | 0.7 |  | Private | 118 | 78.1 |
| Single | 22 | 14.6 |  | Other | 19 | 12.6 |
| Education |  |  |  | Full-time employment | 79 | 52.3 |
| Psychologist | 80 | 53.0 |  | Supervision |  |  |
| Psychiatrist | 5 | 3.3 |  | Once a week | 17 | 11.3 |
| Other | 66 | 43.7 |  | Once a month | 72 | 47.7 |
| Psychotherapeutic approach |  |  |  | Quarterly | 35 | 23.2 |
| Psychodynamic | 27 | 17.9 |  | Less often | 23 | 15.2 |
| CBT | 37 | 24.5 |  | None | 4 | 2.6 |
| Gestalt | 41 | 27.2 |  | Self-experience therapy |  |  |
| Systemic | 36 | 23.8 |  | Finished | 134 | 88.7 |
| Integrative | 20 | 13.2 |  | None | 4 | 2.6 |
| Other | 41 | 27.2 |  | Ongoing | 13 | 8.6 |
| Work experience (mean ± SD) | 15.22 | 9.95 |  | COVID-19-related stress (mean ± SD) | 2.97 | 0.99 |
| Certificate | 132 | 87.4 |  | Online practicing |  |  |
| Weekly workload |  |  |  | Only during the lockdown period | 46 | 30.5 |
| 1–10 h | 25 | 16.6 |  | Currently partially | 84 | 55.6 |
| 11–20 h | 63 | 41.7 |  | Before, during the pandemic, and currently | 15 | 9.9 |
| More than 20 h | 63 | 41.7 |  | Currently only online | 8 | 5.3 |
|  |  |  |  | Only online currently and in the past | 0 | 0.0 |
|  |  |  |  | Stopped working during the pandemic | 4 | 2.6 |
|  |  |  |  | Never | 18 | 11.9 |

Table 2. *Sociodemographic, work-related, and the COVID-19-related stress data of the psychotherapists from Bulgaria (N = 217).*

|  | n | % |  |  | n | % |
| --- | --- | --- | --- | --- | --- | --- |
| Sex |  |  |  | Clients |  |  |
| Male | 24 | 11.1 |  | Children/youth | 122 | 56.2 |
| Female | 193 | 88.9 |  | Adults | 173 | 79.7 |
| Age in years (mean ± SD) | 41.75 | 9.18 |  | Couples | 63 | 29.0 |
| Marital status |  |  |  | Families | 50 | 23.0 |
| Stable relationship | 177 | 81.6 |  | Workplace |  |  |
| Divorced/separated | 20 | 9.2 |  | Public | 21 | 9.7 |
| Widow/widower | 1 | 0.5 |  | Private | 171 | 78.8 |
| Single | 19 | 8.8 |  | Other | 12 | 5.5 |
| Education |  |  |  | Full-time employment | 144 | 66.4 |
| Psychologist | 201 | 92.6 |  | Supervision |  |  |
| Psychiatrist | 4 | 1.8 |  | Once a week | 45 | 20.7 |
| Other | 12 | 5.5 |  | Once a month | 98 | 45.2 |
| Psychotherapeutic approach |  |  |  | Quarterly | 28 | 12.9 |
| Psychodynamic | 65 | 30.0 |  | Less often | 29 | 13.4 |
| CBT | 31 | 14.3 |  | None | 17 | 7.8 |
| Gestalt | 16 | 7.4 |  | Self-experience therapy |  |  |
| Systemic | 42 | 19.4 |  | Finished | 161 | 74.2 |
| Integrative | 16 | 7.4 |  | None | 11 | 5.1 |
| Other | 47 | 21.7 |  | Ongoing | 45 | 20.7 |
| Work experience (mean ± SD) | 9.18 | 8.07 |  | COVID-19-related stress (mean ± SD) | 2.63 | 0.86 |
| Certificate | 121 | 55.8 |  | Online practicing |  |  |
| Weekly workload |  |  |  | Only during the lockdown period | 62 | 28.6 |
| 1–10 h | 113 | 52.1 |  | Currently partially | 87 | 40.1 |
| 11–20 h | 60 | 27.6 |  | Before, during the pandemic, and currently | 40 | 18.4 |
| More than 20 h | 44 | 20.3 |  | Currently only online | 24 | 11.1 |
|  |  |  |  | Only online currently and in the past | 3 | 1.4 |
|  |  |  |  | Stopped working during the pandemic | 13 | 6 |
|  |  |  |  | Never | 0 | 0 |

Table 3. *Sociodemographic, work-related, and the COVID-19-related stress data of the psychotherapists from Cyprus (N = 202).*

|  | n | % |  |  | n | % |
| --- | --- | --- | --- | --- | --- | --- |
| Sex |  |  |  | Clients |  |  |
| Male | 40 | 19.8 |  | Children/youth | 39 | 19.3 |
| Female | 162 | 80.2 |  | Adults | 150 | 74.3 |
| Age in years (mean ± SD) | 37.26 | 9.54 |  | Couples | 10 | 5.0 |
| Marital status |  |  |  | Families | 3 | 1.5 |
| Stable relationship | 131 | 64.9 |  | Workplace |  |  |
| Divorced/separated | 16 | 7.9 |  | Public | 7 | 3.5 |
| Widow/widower | 3 | 1.5 |  | Private | 151 | 74.8 |
| Single | 52 | 25.7 |  | Other | 29 | 14.4 |
| Education |  |  |  | Full-time employment | 125 | 61.9 |
| Psychologist | 173 | 85.6 |  | Supervision |  |  |
| Psychiatrist | 9 | 4.5 |  | Once a week | 43 | 21.3 |
| Other | 20 | 9.9 |  | Once a month | 98 | 48.5 |
| Psychotherapeutic approach |  |  |  | Quarterly | 16 | 7.9 |
| Psychodynamic | 27 | 13.4 |  | Less often | 20 | 9.9 |
| CBT | 70 | 34.7 |  | None | 25 | 12.4 |
| Gestalt | 4 | 2.0 |  | Self-experience therapy |  |  |
| Systemic | 35 | 17.3 |  | Finished | 146 | 72.3 |
| Integrative | 26 | 12.9 |  | None | 21 | 10.4 |
| Other | 40 | 19.8 |  | Ongoing | 35 | 17.3 |
| Work experience (mean ± SD) | 7.89 | 7.01 |  | COVID-19-related stress (mean ± SD) | 3.03 | 1.13 |
| Certificate | 159 | 78.7 |  | Online practicing |  |  |
| Weekly workload |  |  |  | Only during the lockdown period | 59 | 29.2 |
| 1–10 h | 88 | 43.6 |  | Currently partially | 64 | 31.7 |
| 11–20 h | 51 | 25.2 |  | Before, during the pandemic, and currently | 55 | 27.2 |
| More than 20 h | 63 | 31.2 |  | Currently only online | 21 | 10.4 |
|  |  |  |  | Only online currently and in the past | 5 | 2.5 |
|  |  |  |  | Stopped working during the pandemic | 22 | 11 |
|  |  |  |  | Never | 0 | 0 |

Table 4. *Sociodemographic, work-related, and COVID-19-related stress data of the psychotherapists from Finland (N = 254).*

|  | n | % |  |  | n | % |
| --- | --- | --- | --- | --- | --- | --- |
| Sex |  |  |  | Clients |  |  |
| Male | 32 | 12,6 |  | Children/youth | 28 | 11.0 |
| Female | 222 | 87,4 |  | Adults | 169 | 66.5 |
| Age in years (mean ± SD) | 52.65 | 8.73 |  | Couples | 46 | 18.1 |
| Marital status |  |  |  | Families | 11 | 4.3 |
| Stable relationship | 204 | 80.3 |  | Workplace |  |  |
| Divorced/separated | 34 | 13.4 |  | Public | 44 | 17.3 |
| Widow/widower | 4 | 1.6 |  | Private | 192 | 75.6 |
| Single | 12 | 4.7 |  | Other | 18 | 7.1 |
| Education |  |  |  | Full-time employment | 167 | 65.7 |
| Psychologist | 102 | 40.2 |  | Supervision |  |  |
| Psychiatrist | 12 | 4.7 |  | Once a week | 19 | 7.5 |
| Other | 140 | 55.1 |  | Once a month | 190 | 74.8 |
| Psychotherapeutic approach |  |  |  | Quarterly | 28 | 11.0 |
| Psychodynamic | 66 | 26.0 |  | Less often | 9 | 3.5 |
| CBT | 36 | 14.2 |  | None | 8 | 3.1 |
| Gestalt | 2 | 0.8 |  | Self-experience therapy |  |  |
| Systemic | 14 | 5.5 |  | Finished | 213 | 83.9 |
| Integrative | 31 | 12.2 |  | None | 28 | 11.0 |
| Other | 105 | 41.3 |  | Ongoing | 13 | 5.1 |
| Work experience (mean ± SD) | 10.72 | 8.08 |  | COVID-19-related stress (mean ± SD) | 2.68 | 0.87 |
| Certificate | 240 | 94.5 |  | Online practicing |  |  |
| Weekly workload |  |  |  | Only during the lockdown period | 61 | 24.0 |
| 1–10 h | 63 | 24.8 |  | Currently partially | 128 | 50.4 |
| 11–20 h | 78 | 30.7 |  | Before, during the pandemic, and currently | 63 | 24.8 |
| More than 20 h | 113 | 44.5 |  | Currently only online | 0 | 0.0 |
|  |  |  |  | Only online currently and in the past | 6 | 2.4 |
|  |  |  |  | Stopped working during the pandemic | 4 | 1.6 |
|  |  |  |  | Never | 0 | 0 |

Table 5. *Sociodemographic, work-related, and COVID-19-related stress data of the psychotherapists from Spain (N = 320).*

|  | n | % |  |  | n | % |
| --- | --- | --- | --- | --- | --- | --- |
| Sex |  |  |  | Clients |  |  |
| Male | 61 | 19.1 |  | Children/youth | 45 | 14.1 |
| Female | 259 | 80.9 |  | Adults | 151 | 47.2 |
| Age in years (mean ± SD) | 43.47 | 10.90 |  | Couples | 13 | 4.1 |
| Marital status |  |  |  | Families | 2 | 0.6 |
| Stable relationship | 218 | 68.1 |  | Workplace |  |  |
| Divorced/separated | 39 | 12.2 |  | Public | 17 | 5.3 |
| Widow/widower | 1 | 0.3 |  | Private | 225 | 70.3 |
| Single | 62 | 19.4 |  | Other | 82 | 25.6 |
| Education |  |  |  | Full-time employment | 214 | 66.9 |
| Psychologist | 320 | 100.0 |  | Supervision |  |  |
| Psychiatrist | 0 | 0.0 |  | Once a week | 55 | 17.2 |
| Other | 0 | 0 |  | Once a month | 84 | 26.3 |
| Psychotherapeutic approach |  |  |  | Quarterly | 32 | 10.0 |
| Psychodynamic | 30 | 9.4 |  | Less often | 38 | 11.9 |
| CBT | 158 | 49.4 |  | None | 111 | 34.7 |
| Gestalt | 14 | 4.4 |  | Self-experience therapy |  |  |
| Systemic | 39 | 12.2 |  | Finished | 210 | 65.6 |
| Integrative | 59 | 18.4 |  | None | 81 | 25.3 |
| Other | 0 | 0.0 |  | Ongoing | 29 | 9.1 |
| Work experience (mean ± SD) | 13.18 | 9.25 |  | COVID-19-related stress (mean ± SD) | 3.07 | 1.09 |
| Certificate | 280 | 87.5 |  | Online practicing |  |  |
| Weekly workload |  |  |  | Only during the lockdown period | 64 | 20.0 |
| 1–10 h | 87 | 27.2 |  | Currently partially | 86 | 26.9 |
| 11–20 h | 116 | 36.3 |  | Before, during the pandemic, and currently | 91 | 28.4 |
| More than 20 h | 117 | 36.6 |  | Currently only online | 23 | 7.2 |
|  |  |  |  | Only online currently and in the past | 1 | 0.3 |
|  |  |  |  | Stopped working during the pandemic | 30 | 9.4 |
|  |  |  |  | Never | 0 | 0 |

Table 6. *Sociodemographic, work-related, and COVID-19-related stress data of the psychotherapists from Norway (N = 225).*

|  | n | % |  |  | n | % |
| --- | --- | --- | --- | --- | --- | --- |
| Sex |  |  |  | Clients |  |  |
| Male | 55 | 24.4 |  | Children/youth | 60 | 26.7 |
| Female | 170 | 75.6 |  | Adults | 209 | 92.9 |
| Age in years (mean ± SD) | 53.58 | 10.05 |  | Couples | 31 | 13.8 |
| Marital status |  |  |  | Families | 34 | 15.1 |
| Stable relationship | 178 | 79.1 |  | Workplace |  |  |
| Divorced/separated | 22 | 9.8 |  | Public | 72 | 32.0 |
| Widow/widower | 3 | 1.3 |  | Private | 124 | 55.1 |
| Single | 22 | 9.8 |  | Other | 38 | 16.9 |
| Education |  |  |  | Full-time employment | 161 | 71.6 |
| Psychologist | 112 | 49.8 |  | Supervision |  |  |
| Psychiatrist | 37 | 16.4 |  | Once a week | 41 | 18.2 |
| Other | 37 | 16 |  | Once a month | 72 | 32.0 |
| Psychotherapeutic approach |  |  |  | Quarterly | 24 | 10.7 |
| Psychodynamic | 108 | 48.0 |  | Less often | 28 | 12.4 |
| CBT | 81 | 36.0 |  | None | 60 | 26.7 |
| Gestalt | 20 | 8.9 |  | Self-experience therapy |  |  |
| Systemic | 14 | 6.2 |  | Finished | 189 | 84.0 |
| Integrative | 18 | 8.0 |  | None | 10 | 4.4 |
| Other | 59 | 26.2 |  | Ongoing | 26 | 11.6 |
| Work experience (mean ± SD) | 17.92 | 10.91 |  | COVID-19-related stress (mean ± SD) | 2.64 | 0.98 |
| Certificate | 191 | 84.9 |  | Online practicing |  |  |
| Weekly workload |  |  |  | Only during the lockdown period | 84 | 37.3 |
| 1–10 h | 50 | 22.2 |  | Currently partially | 110 | 48.9 |
| 11–20 h | 53 | 23.6 |  | Before, during the pandemic, and currently | 23 | 10.2 |
| More than 20 h | 122 | 54.2 |  | Currently only online | 18 | 8.0 |
|  |  |  |  | Only online currently and in the past | 11 | 4.9 |
|  |  |  |  | Stopped working during the pandemic | 5 | 2.2 |
|  |  |  |  | Never | 0 | 0 |

Table 7. *Sociodemographic, work-related, and COVID-19-related stress data of the psychotherapists from Poland (N = 340).*

|  | n | % |  |  | n | % |
| --- | --- | --- | --- | --- | --- | --- |
| Sex |  |  |  | Clients |  |  |
| Male | 43 | 12.6 |  | Children/youth | 123 | 36.2 |
| Female | 297 | 87.4 |  | Adults | 320 | 94.1 |
| Age in years (mean ± SD) | 40.71 | 7.99 |  | Couples | 104 | 30.6 |
| Marital status |  |  |  | Families | 64 | 18.8 |
| Stable relationship | 265 | 77.9 |  | Workplace |  |  |
| Divorced/separated | 29 | 8.5 |  | Public | 66 | 19.4 |
| Widow/widower | 6 | 1.8 |  | Private | 276 | 81.2 |
| Single | 40 | 11.8 |  | Other | 18 | 5.3 |
| Education |  |  |  | Full-time employment | 288 | 84.7 |
| Psychologist | 251 | 73.8 |  | Supervision |  |  |
| Psychiatrist | 6 | 1.8 |  | Once a week | 83 | 24.4 |
| Other | 83 | 24.4 |  | Once a month | 223 | 65.6 |
| Psychotherapeutic approach |  |  |  | Quarterly | 19 | 5.6 |
| Psychodynamic | 66 | 19.4 |  | Less often | 11 | 3.2 |
| CBT | 80 | 23.5 |  | None | 4 | 1.2 |
| Gestalt | 70 | 20.6 |  | Self-experience therapy |  |  |
| Systemic | 38 | 11.2 |  | Finished | 254 | 74.7 |
| Integrative | 72 | 21.2 |  | None | 13 | 3.8 |
| Other | 62 | 18.2 |  | Ongoing | 73 | 21.5 |
| Work experience (mean ± SD) | 8.46 | 6.71 |  | COVID-19-related stress (mean ± SD) | 3.30 | 1.04 |
| Certificate | 129 | 37.9 |  | Online practicing |  |  |
| Weekly workload |  |  |  | Only during the lockdown period | 98 | 28.8 |
| 1–10 h | 72 | 21.2 |  | Currently partially | 181 | 53.2 |
| 11–20 h | 120 | 35.3 |  | Before, during the pandemic, and currently | 59 | 17.4 |
| More than 20 h | 148 | 43.5 |  | Currently only online | 39 | 11.5 |
|  |  |  |  | Only online currently and in the past | 5 | 1.5 |
|  |  |  |  | Stopped working during the pandemic | 9 | 2.6 |
|  |  |  |  | Never | 0 | 0 |

Table 8. *Sociodemographic, work-related, and COVID-19-related stress data of the psychotherapists from Romania (N = 202).*

|  | n | % |  |  | n | % |
| --- | --- | --- | --- | --- | --- | --- |
| Sex |  |  |  | Clients |  |  |
| Male | 25 | 12.4 |  | Children/youth | 96 | 47.5 |
| Female | 177 | 87.6 |  | Adults | 178 | 88.1 |
| Age in years (mean ± SD) | 37.78 | 10.15 |  | Couples | 36 | 17.8 |
| Marital status |  |  |  | Families | 49 | 24.3 |
| Stable relationship | 153 | 75.7 |  | Workplace |  |  |
| Divorced/separated | 12 | 5.9 |  | Public | 42 | 20.8 |
| Widow/widower | 14 | 6.9 |  | Private | 177 | 87.6 |
| Single | 1 | 0.5 |  | Other | 4 | 2.0 |
| Education |  |  |  | Full-time employment | 114 | 56.4 |
| Psychologist | 185 | 91.6 |  | Supervision |  |  |
| Psychiatrist | 5 | 2.5 |  | Once a week | 39 | 19.3 |
| Other | 12 | 5.9 |  | Once a month | 64 | 31.7 |
| Psychotherapeutic approach |  |  |  | Quarterly | 30 | 14.9 |
| Psychodynamic | 43 | 21.3 |  | Less often | 42 | 20.8 |
| CBT | 86 | 42.6 |  | None | 27 | 13.4 |
| Gestalt | 2 | 1.0 |  | Self-experience therapy |  |  |
| Systemic | 16 | 7.9 |  | Finished | 147 | 72.8 |
| Integrative | 31 | 15.3 |  | None | 14 | 6.9 |
| Other | 53 | 26.2 |  | Ongoing | 41 | 20.3 |
| Work experience (mean ± SD) | 6.86 | 6.78 |  | COVID-19-related stress (mean ± SD) | 3.00 | 1.00 |
| Certificate | 167 | 82.7 |  | Online practicing |  |  |
| Weekly workload |  |  |  | Only during the lockdown period | 42 | 20.8 |
| 1–10 h | 96 | 47.5 |  | Currently partially | 75 | 37.1 |
| 11–20 h | 46 | 22.8 |  | Before, during the pandemic, and currently | 47 | 23.3 |
| More than 20 h | 60 | 29.7 |  | Currently only online | 63 | 31.2 |
|  |  |  |  | Only online currently and in the past | 1 | 0.5 |
|  |  |  |  | Stopped working during the pandemic | 19 | 9.4 |
|  |  |  |  | Never | 0 | 0 |

Table 9. *Sociodemographic, work-related, and COVID-19-related stress data of the psychotherapists from Serbia (N = 237).*

|  | n | % |  |  | n | % |
| --- | --- | --- | --- | --- | --- | --- |
| Sex |  |  |  | Clients |  |  |
| Male | 32 | 13.5 |  | Children/youth | 52 | 21.9 |
| Female | 205 | 86.5 |  | Adults | 179 | 75.5 |
| Age in years (mean ± SD) | 39.28 | 9.72 |  | Couples | 4 | 1.7 |
| Marital status |  |  |  | Families | 2 | 0.8 |
| Stable relationship | 168 | 70.9 |  | Workplace |  |  |
| Divorced/separated | 25 | 10.5 |  | Public | 12 | 5.1 |
| Widow/widower | 2 | 0.8 |  | Private | 192 | 81.0 |
| Single | 42 | 17.7 |  | Other | 11 | 4.6 |
| Education |  |  |  | Full-time employment | 83 | 35.0 |
| Psychologist | 174 | 73.4 |  | Supervision |  |  |
| Psychiatrist | 17 | 7.2 |  | Once a week | 21 | 8.9 |
| Other | 46 | 19.4 |  | Once a month | 88 | 37.1 |
| Psychotherapeutic approach |  |  |  | Quarterly | 38 | 16.0 |
| Psychodynamic | 45 | 19.0 |  | Less often | 55 | 23.2 |
| CBT | 40 | 16.9 |  | None | 35 | 14.8 |
| Gestalt | 26 | 11.0 |  | Self-experience therapy |  |  |
| Systemic | 23 | 9.7 |  | Finished | 157 | 66.2 |
| Integrative | 48 | 20.3 |  | None | 9 | 3.8 |
| Other | 55 | 23.2 |  | Ongoing | 71 | 30.0 |
| Work experience (mean ± SD) | 8.31 | 7.38 |  | COVID-19-related stress (mean ± SD) | 2.84 | 0.83 |
| Certificate | 160 | 67.5 |  | Online practicing |  |  |
| Weekly workload |  |  |  | Only during the lockdown period | 54 | 22.8 |
| 1–10 h | 140 | 59.1 |  | Currently partially | 78 | 32.9 |
| 11–20 h | 58 | 24.5 |  | Before, during the pandemic, and currently | 45 | 19.0 |
| More than 20 h | 39 | 16.5 |  | Currently only online | 31 | 13.1 |
|  |  |  |  | Only online currently and in the past | 16 | 6.8 |
|  |  |  |  | Stopped working during the pandemic | 13 | 5.5 |
|  |  |  |  | Never | 0 | 0 |

Table 10. *Sociodemographic, work-related, and COVID-19-related stress data of the psychotherapists from Switzerland (N = 205).*

|  | n | % |  |  | n | % |
| --- | --- | --- | --- | --- | --- | --- |
| Sex |  |  |  | Clients |  |  |
| Male | 47 | 22.9 |  | Children/youth | 87 | 42.4 |
| Female | 158 | 77.1 |  | Adults | 198 | 96.6 |
| Age in years (mean ± SD) | 50.16 | 11.13 |  | Couples | 55 | 26.8 |
| Marital status |  |  |  | Families | 77 | 37.6 |
| Stable relationship | 156 | 76.1 |  | Workplace |  |  |
| Divorced/separated | 23 | 11.2 |  | Public | 0 | 0.0 |
| Widow/widower | 6 | 2.9 |  | Private | 0 | 0.0 |
| Single | 20 | 9.8 |  | Other | 0 | 0.0 |
| Education |  |  |  | Full-time employment | 90 | 43.9 |
| Psychologist | 161 | 78.5 |  | Supervision |  |  |
| Psychiatrist | 5 | 2.4 |  | Once a week | 16 | 7.8 |
| Other | 39 | 19.0 |  | Once a month | 112 | 54.6 |
| Psychotherapeutic approach |  |  |  | Quarterly | 48 | 23.4 |
| Psychodynamic | 51 | 24.9 |  | Less often | 23 | 11.2 |
| CBT | 32 | 15.6 |  | None | 6 | 2.9 |
| Gestalt | 62 | 30.2 |  | Self-experience therapy |  |  |
| Systemic | 42 | 20.5 |  | Finished | 165 | 80.5 |
| Integrative | 39 | 19.0 |  | None | 10 | 4.9 |
| Other | 54 | 26.3 |  | Ongoing | 30 | 14.6 |
| Work experience (mean ± SD) | 15.12 | 10.27 |  | COVID-19-related stress (mean ± SD) | 3.07 | 1.03 |
| Certificate | 165 | 80.5 |  | Online practicing |  |  |
| Weekly workload |  |  |  | Only during the lockdown period | 67 | 32.7 |
| 1–10 h | 39 | 19.0 |  | Currently partially | 108 | 52.7 |
| 11–20 h | 80 | 39.0 |  | Before, during the pandemic, and currently | 16 | 7.8 |
| More than 20 h | 86 | 42.0 |  | Currently only online | 4 | 2.0 |
|  |  |  |  | Only online currently and in the past | 0 | 0 |
|  |  |  |  | Stopped working during the pandemic | 6 | 2.9 |
|  |  |  |  | Never | 29 | 14.1 |

Table 11. *Sociodemographic, work-related, and COVID-19-related stress data of the psychotherapists from Sweden (N = 275).*

|  | n | % |  |  | n | % |
| --- | --- | --- | --- | --- | --- | --- |
| Sex |  |  |  | Clients |  |  |
| Male | 59 | 21.5 |  | Children/youth | 36 | 13.1 |
| Female | 216 | 78.5 |  | Adults | 220 | 80.0 |
| Age in years (mean ± SD) | 48.99 | 11.44 |  | Couples | 9 | 3.3 |
| Marital status |  |  |  | Families | 10 | 3.6 |
| Stable relationship | 223 | 81.1 |  | Workplace |  |  |
| Divorced/separated | 5 | 1.8 |  | Public | 129 | 46.9 |
| Widow/widower | 2 | 0.7 |  | Private | 123 | 44.7 |
| Single | 45 | 16.4 |  | Other | 9 | 3.3 |
| Education |  |  |  | Full-time employment | 143 | 52.0 |
| Psychologist | 143 | 52.0 |  | Supervision |  |  |
| Psychiatrist | 2 | 0.7 |  | Once a week | 26 | 9.5 |
| Other | 130 | 47.3 |  | Once a month | 166 | 60.4 |
| Psychotherapeutic approach |  |  |  | Quarterly | 32 | 11.6 |
| Psychodynamic | 134 | 48.7 |  | Less often | 34 | 12.4 |
| CBT | 66 | 24.0 |  | None | 17 | 6.2 |
| Gestalt | 0 | 0.0 |  | Self-experience therapy |  |  |
| Systemic | 15 | 5.5 |  | Finished | - | - |
| Integrative | 53 | 19.3 |  | None | - | - |
| Other | 7 | 2.5 |  | Ongoing | - | - |
| Work experience (mean ± SD) | 12.45 | 8.88 |  | COVID-19-related stress (mean ± SD) | 2.33 | 0.91 |
| Certificate | 135 | 49.1 |  | Online practicing |  |  |
| Weekly workload |  |  |  | Only during the lockdown period | 46 | 16.7 |
| 1–10 h | 83 | 30.2 |  | Currently partially | 152 | 55.3 |
| 11–20 h | 132 | 48.0 |  | Before, during the pandemic, and currently | 28 | 10.2 |
| More than 20 h | 60 | 21.8 |  | Currently only online | 17 | 6.2 |
|  |  |  |  | Only online currently and in the past | 0 | 0.0 |
|  |  |  |  | Stopped working during the pandemic | 2 | 0.7 |
|  |  |  |  | Never | 41 | 14.9 |

Table 12. *Sociodemographic, work-related, and COVID-19-related stress data of the psychotherapists from the United Kingdom (N = 287).*

|  | n | % |  |  | n | % |
| --- | --- | --- | --- | --- | --- | --- |
| Sex |  |  |  | Clients |  |  |
| Male | 47 | 16.4 |  | Children/youth | 70 | 24.4 |
| Female | 240 | 83.6 |  | Adults | 256 | 89.2 |
| Age in years (mean ± SD) | 50.26 | 12.06 |  | Couples | 34 | 11.8 |
| Marital status |  |  |  | Families | 56 | 19.5 |
| Stable relationship | 206 | 71.8 |  | Workplace |  |  |
| Divorced/separated | 38 | 13.2 |  | Public | 74 | 25.8 |
| Widow/widower | 2 | 0.7 |  | Private | 180 | 62.7 |
| Single | 41 | 14.3 |  | Other | 66 | 23.0 |
| Education |  |  |  | Full-time employment | 175 | 61.0 |
| Psychologist | 256 | 89 |  | Supervision |  |  |
| Psychiatrist | 0 | 0 |  | Once a week | 86 | 30.0 |
| Other | 31 | 11 |  | Once a month | 189 | 65.9 |
| Psychotherapeutic approach |  |  |  | Quarterly | 7 | 2.4 |
| Psychodynamic | 64 | 22.3 |  | Less often | 3 | 1.0 |
| CBT | 61 | 21.3 |  | None | 2 | 0.7 |
| Gestalt | 11 | 3.8 |  | Self-experience therapy |  |  |
| Systemic | 13 | 4.5 |  | Finished | 228 | 79.4 |
| Integrative | 150 | 52.3 |  | None | 46 | 16.0 |
| Other | 74 | 25.8 |  | Ongoing | 13 | 4.5 |
| Work experience (mean ± SD) | 12.79 | 9.44 |  | COVID-19-related stress (mean ± SD) | 3.33 | 1.09 |
| Certificate | 250 | 87.1 |  | Online practicing |  |  |
| Weekly workload |  |  |  | Only during the lockdown period | 70 | 24.4 |
| 1–10 h | 101 | 35.2 |  | Currently partially | 86 | 30.0 |
| 11–20 h | 116 | 40.4 |  | Before, during the pandemic, and currently | 25 | 8.7 |
| More than 20 h | 70 | 24.4 |  | Currently only online | 129 | 44.9 |
|  |  |  |  | Only online currently and in the past | 0 | 0.0 |
|  |  |  |  | Stopped working during the pandemic | 1 | 0.3 |
|  |  |  |  | Never | 1 | 0.3 |


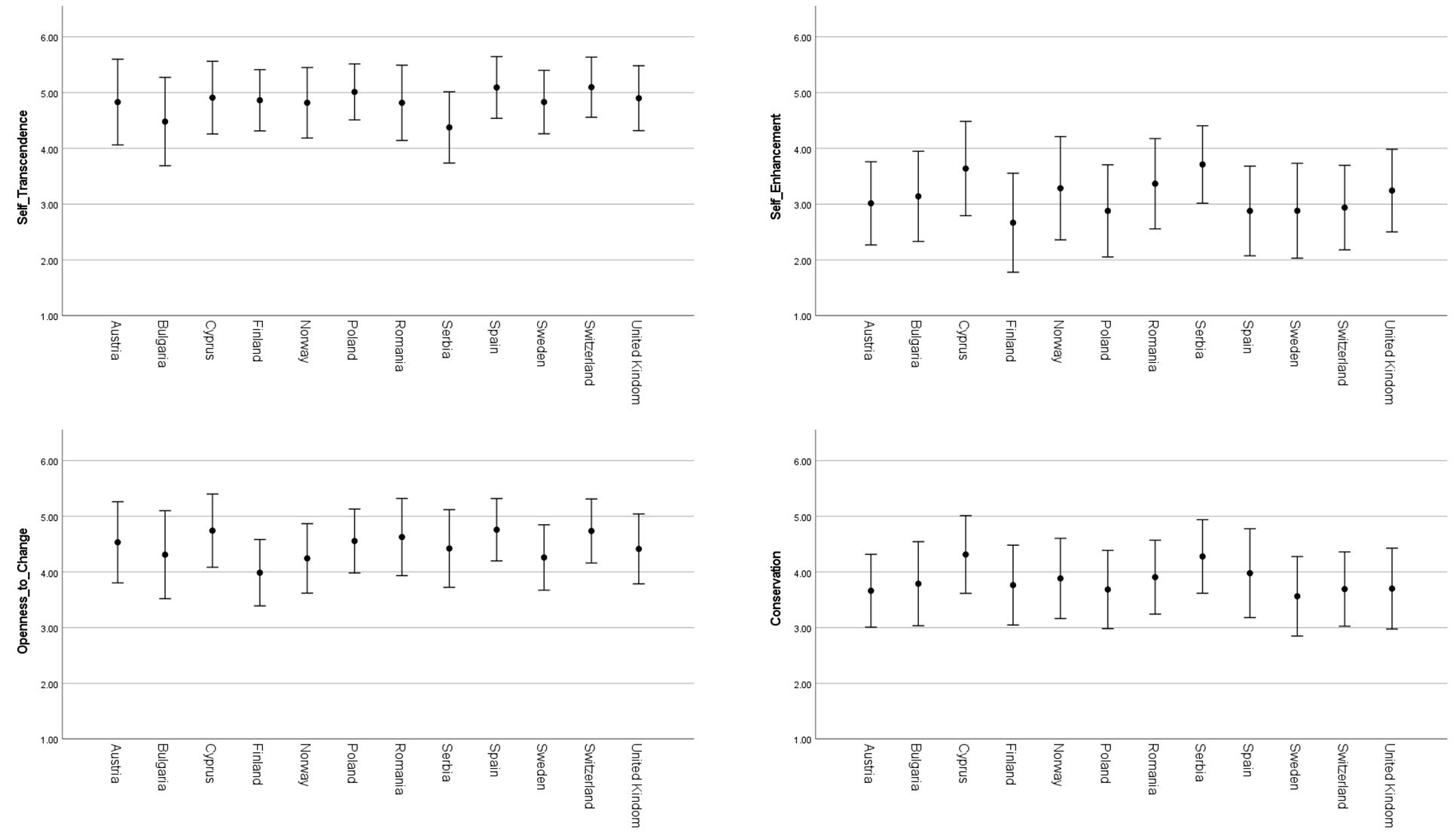


Figure 1S. Cultural values: Country-level means with plus/minus 1 standard deviation.


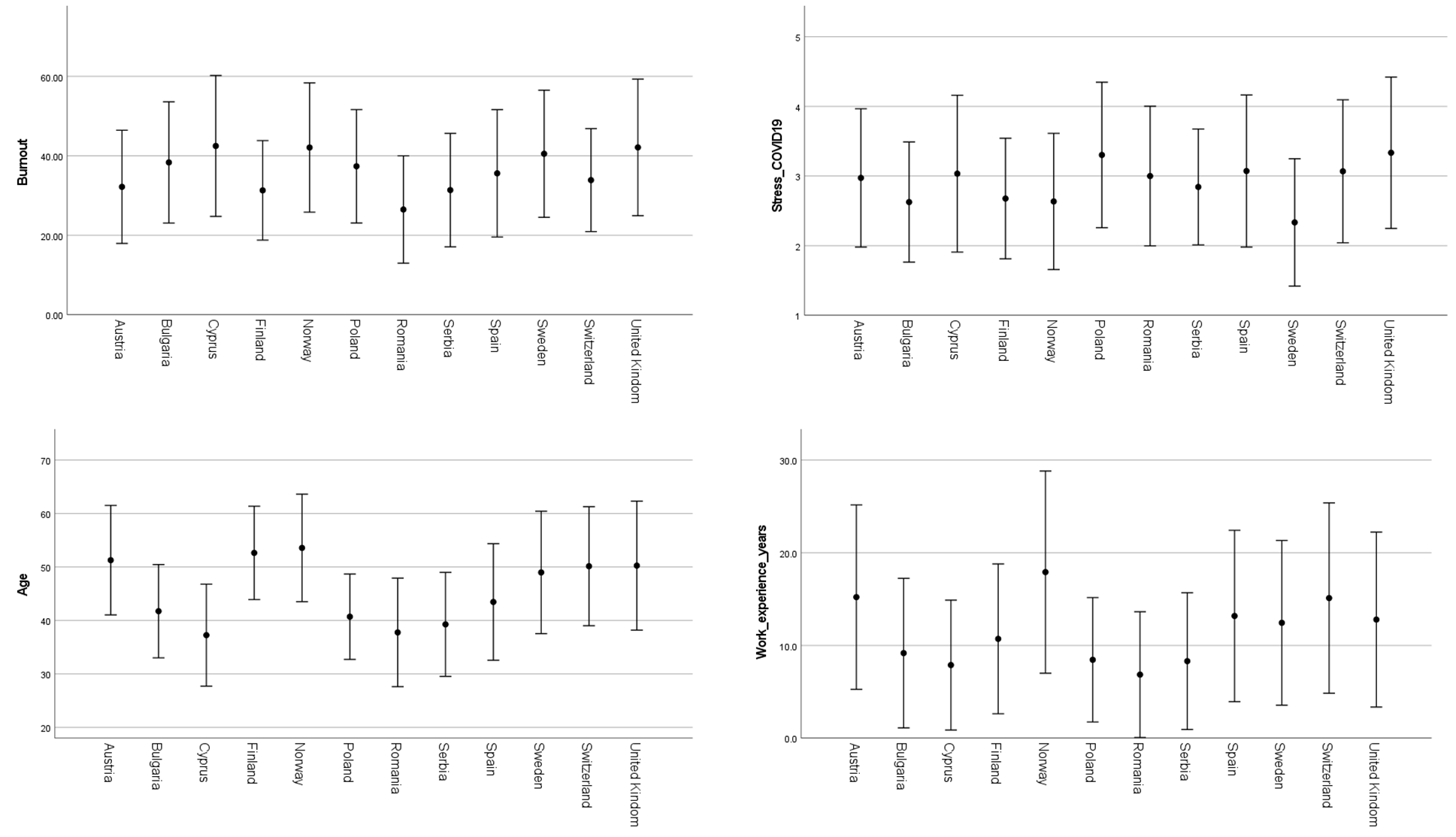


Figure 2S. Burnout, COVID19-related stress, Age and Work experience: Country-level means with plus/minus 1 standard deviation.


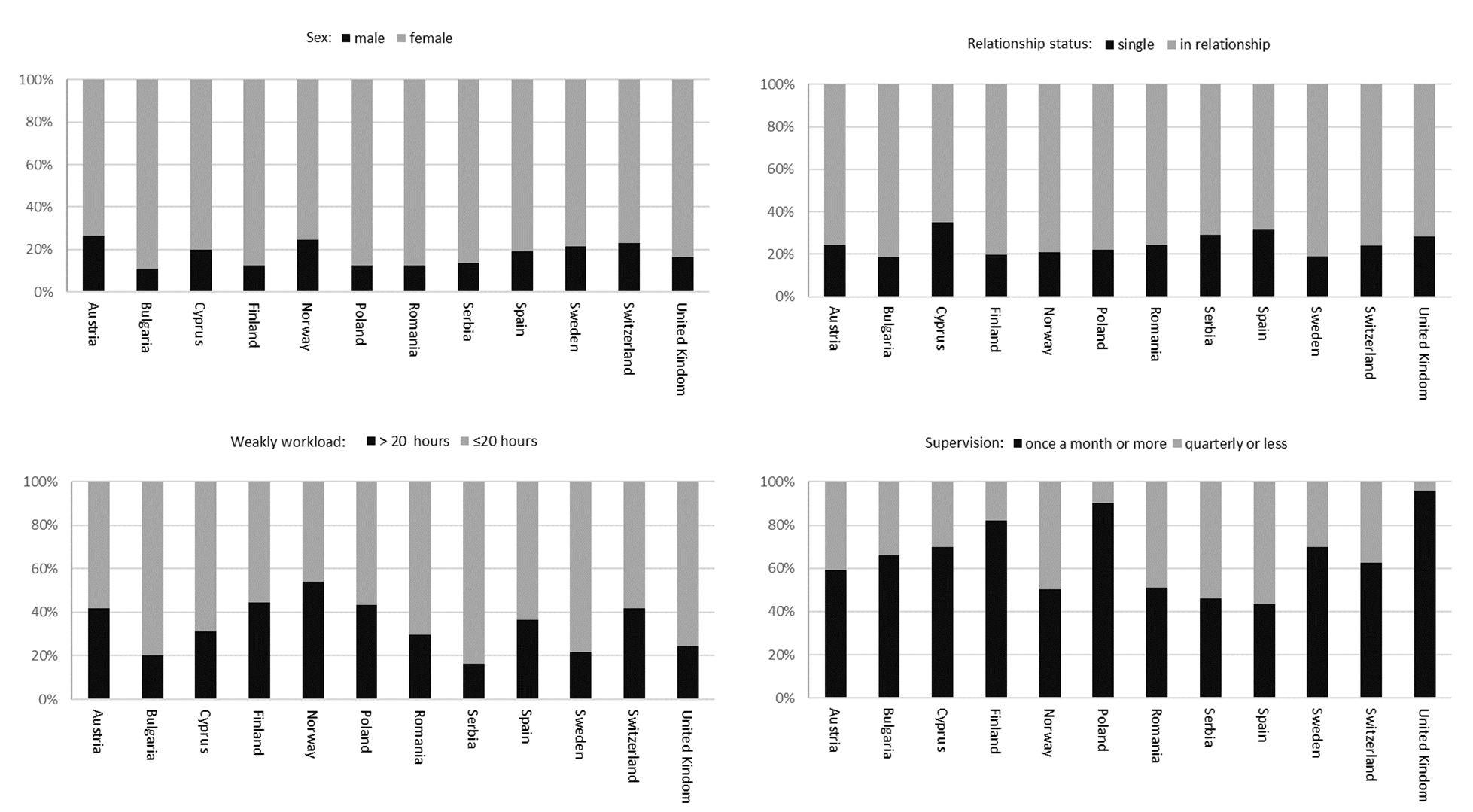


Figure 3S. Sex, Relationship status, Weakly workload and Supervision frequency: Country-level proportions.
